# Midwifery students' experiences of support for ethical competence

**DOI:** 10.1177/0969733021999773

**Published:** 2021-08-27

**Authors:** Leena Honkavuo

**Affiliations:** Åbo Akademi University, Finland

**Keywords:** Challenging situations, ethical competence, experiences, hermeneutics, midwifery student, support

## Abstract

**Background:**

Midwifery students are confronted with several ethical dilemmas and challenging situations during clinical midwifery care practice. Since ethical competence of midwifery students is under development, it is important to support the students’ learning progress of ethical issues from diverse viewpoints.

**Objective:**

From the perspective of didactics of caring science and the context of midwifery students, to explore how midwifery students’ experience supports for ethical competence in midwifery education and investigate how ethically challenging situations have been carried out during clinical midwifery care practice.

**Design:**

Qualitative, explorative and descriptive design with inductive nature.

**Methods:**

Focus group interviews with nine Swedish midwifery students. Hans-Georg Gadamer’s philosophical hermeneutics was applied to guide the interpretation.

**Ethical considerations:**

Ethical principles and scientific guidelines were followed. Informed consent was obtained from the participants. Confidentiality was respected and quotations anonymised.

**Results:**

Receiving support when ethically challenging situations occur in clinical midwifery practice is important and necessary. One main theme, such as support is a human and caring factor in the midwifery students’ Bildung process on ethical competence, and four subthemes, such as supporting through trust and responsibility; supporting through dignity and respect; supporting through truthfulness and justice; and supporting through dialogue and reflection, were created from the hermeneutical interpretation.

**Discussion:**

Teaching ethics should be carefully planned, consistent and continue throughout the midwifery education. There is dispersion in the pedagogy of ethical situations, the methods and perceptions associated with it, and in obtaining possible support for students. Developing well-experienced methods could benefit the support of midwifery students’ ethical competence when they experience ethically challenging situations in midwifery care practice.

## Introduction

The diversity of ethical challenges has increased in the past years in midwifery care. The change is due to the development of in vitro treatments and foetal diagnostics, the rising age of first-time mothers, pregnant women’s chronical health issues and the multipled number of several drug users; as a result of the immigration from third countries, linguistic challenges connected with cultural and religious traditions and values arise.^1–3^

In the ethics of midwifery care, the midwife is obliged to simultaneously consider the needs of several individuals, although the basic features of the ethics of caring are similar to those of nursing care.^2–4^ The ethics is governed by current legislation, general instructions, guidelines and codes. It is based on dignity, understanding and respecting human beings and their rights to care.^3,4^ In Sweden, the midwifery students are Registered Nurses with 180 European Credit Transfer System (ECTS). The midwifery programme comprises 90 ECTS and consists of two equal parts with 45 ECTS theoretical and 45 ECTS clinical-based practice. The total study time is 4.5 years.^3^

The concept *competence* origins from Ancient Greece and Plato’s philosophy.^5,6^ Benner^7^ introduced *competence* as a concept in clinical nursing practice in 1984. Since then competence is used in nursing and caring sciences to describe the caregiver’s capability to function in nursing settings to encourage and support holistically the patient’s health and alleviate suffering. Competence is connected to professionality, proficiency, quality, evidence-based knowledge and critical thinking, experience and motivation.^2,4,7,8^ Competence is an outcome of professional ethics and influenced by historical and current impacts in the context of midwifery care. Ethical competence is connected to the midwife student’s basic ethical value base ethos, and inner moral skills to identify disagreements of values and ethical dimensions, to prioritise and choose values that are grounded on logical reasoning and to function, based on the decisions that have been taken.^9–13^ Ethical competence allows midwife students to make ethically complicated and difficult decisions, and to implement ethically defendable clinical midwifery care practice.^1,2,4,11,14,15^

This study’s theoretical perspective is anchored in Matilainen and Eriksson’s^16^ humanistic caring science tradition and the didactics of caring science with a hermeneutic formation ideal. The didactics of caring science is developed from pedagogy into a subdiscipline in caring science. It is based on the same ontology, epistemology, theoretical starting point and ethos.^9,10,16^ Ontology opens for entities attributed to support and supervision of midwifery student in clinical midwifery care practice. Implicitly, the didactics of caring science, art and culture create a synthesis representing the ethical foundation for the ethos of caring knowledge and its inner value pattern. This formation portrays the conditions for the creation of a culture of the didactics of caring science that enables growth and becoming. Ethics motivates and involves for dignity of the human beings. Ethics leads the ontology in theory and in clinical practice. Ontology implies that midwifery students embrace an attitude to the clinical midwifery care context they influence through holistic caring.^9,10^

The study’s epistemological and methodological starting point is in human sciences and Gadamer’s^17^ hermeneutical philosophy. The pre-understanding as the affiliation to a caring science tradition and the midwifery students’ context is important in hermeneutical philosophy. Gadamer^17^ uses the educational concept *Bildung* to describe an inner identity for formation of becoming a professional midwife. In this study, *Bildung* represents the search for truth and critical thinking and the ability to evaluate, consider and change perceptions and behaviours that are based on a better understanding of the context.^17^ It includes an aesthetic ability of ethical sense. Bildung is integrated into midwifery students’ personality and ethos that serves him or her as a motivation and driving force. Bildung becomes visible through formation and when the ethos is dedicated to the human beings themselves.^17^ This means that ethos and ethics are merged to one, when theory is transferred into ethos and carried out through midwifery care practice. The German scientist von Humboldt (1767–1835) explains according to formation in higher education that Bildung is an intellectual investigation of humanistic ideals, and that theory should be guided by existing research and science. Formation is connected to cultivation, academia, freedom, free will, solitude and the intellect.^19^ It becomes visible through affects, action and bearing in the context of clinical midwifery care.

## Background

Facing ethical dilemmas is associated with ethical decision-making and the support of midwifery students; ethical decision-making requires ethical sensitivity, which means the ability to both identify ethical dilemmas and consider the implications of the midwifery students’ actions for others.^1,11,13,19^ Ethical motivation is important so that the midwifery students can commit to activities in accordance with ethical values and take responsibility for the consequences. Moral intensity associated with an ethical challenge affects all stages of ethical decision-making in clinical midwifery care practice.^1,2,9,13,19,20^

Ethics and ethical competence has been investigated from the perspective of nursing care with focus on nurses and nursing students; thus, the codes of ethics are, in principle, used by all kinds of healthcare professionals worldwide.^2,4,21,22^ The importance of ethical issues in clinical midwifery care practice has been centred to the viewpoint of supervision. Supporting ethical competence in midwifery students has significantly got less focus as well as the discussion about ethically challenging situations in midwifery care.

Supervised clinical midwifery care practice is important for midwifery student’s growth and development.^1,2,23–25^ A positive and effective period at the clinic or healthcare organisation strengthens self-esteem, enables the combination of theoretical knowledge and practice, increases the experience of qualifications, and develops the student’s professional identity.^12,22,23^ Ethical competence is connected to character building and related to the Aristotelian virtues such as being truthful, empathic and dedicated to the patient’s situation. Ethical competence is possible to be applicated through good role models, living examples and clinical practice experience.^4,6,11–13,21^ Thus, ethical guidelines are essential in all healthcare; according to Höglund et al.,^26^ they have not been identified as beneficial in the midwifery students’ learning process.

Midwifery students meet adolescents and women of different ages in various, ethically challenging situations.^3^ Continuous reflection on ethical principles can be a strategy and an approach to handle difficult ethical challenges during clinical midwifery care practice.^13,14,23,27,28^ Pregnancy abruptions require ethical skills and ethical sensitivity and are associated with a wide range of ethical issues related to the age and health of the embryo and the woman, and woman’s value base. The woman’s family situation and the ability to take care of the newborn child may cause obstacles.^28–30, 32^ An ethical problem arises when pregnancy would be a health risk for the woman and her life. Methods to protect the health of the foetus can sometimes conflict with the pregnant woman’s autonomy or choices in life. The use of drugs, alcohol or medicines and social or other pregnancy-related problems that may cause permanent injury to the developing foetus cause the society ethical challenges.^27–31^

Perceptions of the right to life pose ethical difficulties among many students.^14,29^ The birth of a premature child and to that associated obstetric and paediatric medical complications and treatments can as well cause several ethically challenging experiences.^3,28,30^ Infertility treatments often involve both physical and mental suffering and ethical dilemmas.^28,30,32^ Genetic research related to the beginning and end of life and foetal diagnosis has increased ethical conflict situations over the last decades.^28,30,31^ It is challenging when abnormalities are detected during the routine ultrasound examination.^32^ Some pregnancies end with intrauterine foetal death or a newborn with serious health problems.^27,28,30,32^ Women with hormone disorders, gynaecological diseases and cancer as well as urinary tract problems are assisted by midwifery students.^31^ Different traditions, cultures and religious beliefs cause challenging ethical reflections related to prevention, pregnancy and childbirth.^3,27,28,30^

Supervision and midwifery education is studied for decades, through different viewpoints and research methods.^2,23^ Previous studies have drawn attention to clinical and practical midwifery interventions, clinical issues and methods. There is a need to explore cross-cultural, longitudinal, ethnographic and quantitative studies on challenging ethics situations in midwifery education and clinical midwifery care practice that take account on European educational standards.

Pedagogical education and clinical supervision is formed from the midwifery students’ perspective based on the didactics of caring science. The support for enhanced ethical competence and ethically challenging situations in the midwifery student context has previously got less focus in caring science research, but the knowledge of support exists in didactics and pedagogical science. The study seeks to answer the following research questions:

**Research Question 1:** What are the midwifery students’ experiences on support for ethical competence during midwifery education in clinical midwifery care practice?
**Research Question 2:** How have the ethically challenging situations been carried out during clinical midwifery care practice?

## Methods

The study method follows Gadamerian hermeneutics according to knowledge-seeking questions, pre-understanding and prejudices, about the nature of understanding and interpretation as whole, and at the same time is situated within Eriksson’s caring science tradition.^9,10,16,17^ In this study, the seeking for knowledge implies that ethics leads ontology and involves attitudes and assumptions that are based on ethos, dignity and responsibility.^9^ The study’s pre-understanding and prejudices are based on the researcher’s knowledge and experience from the clinical field of midwifery care as well as the didactics of caring science and research, encountering the caring of human beings.^33^

The design is qualitative, explorative and descriptive and has an inductive character. The nature of exploration is open to support the achievement of the study object. The empirical data are collected inductively from empirical findings of the text and the focus group interviews of midwifery students in clinical midwifery practice.^33,34^ An inductive approach allows for the description and interpretation of the midwifery students and their lifeworld. The inductive approach offers explanations for empirical variation.^33^ The language in the hermeneutic semi-structured interviews represents description, expression, understanding and interpretation.^17^

### Data collection and participants

The data were collected in Sweden at three educational institutions at the university college level that educate midwives. A written request to conduct the study was responded positively by the educational institutions. The choice to use these institutions was strategic and based on the researchers’ hermeneutic understanding of the topic. The participants were recruited through purposive selection.^33–35^

In semi-structured focus group interviews, nine midwifery students on the second semester of midwifery studies participated and related experiences of support and supervision according to ethical competence in midwifery education. Each focus group consisted of three participants. Based on the study’s methodology and a pre-tested semi-structured interview guide with two midwifery students, there was a reason to assume that the number of participants and focus groups was sufficient to reach saturation.^33,34,36^

Inclusion criteria for the selection of participants were that they were at least second-semester midwifery students and had knowledge of clinical midwifery care in theory, practice and caring science. Caring science was mainly related to Eriksson’s caring science tradition; thus, the participants were also familiar with other caring science and nursing theories from the first year in nurse-midwifery education. Each participant had to have personal experiences of challenging ethical situations in clinical midwifery care practice. Emphasis was placed on the exclusion criteria so that the results would not be inaccurate. Participants with less than 1 year of midwifery studies and participants with less than two clinical field periods from midwifery care practice were excluded. All participants were females. The interviews were initiated by collecting demographic data. The age of the participants was between 23 and 33 years (mean age: 26.5 years). Two of the participants had earlier education from other disciplines than healthcare. The participants had the similar level of socioeconomic backgrounds, and they all had equal caring science competence due to theoretical midwifery education and clinical practice. The participants were encouraged to share their lived experiences in relation to the provision and receipt of clinical midwifery care practice, respectively. They were invited to the study with an initial question: ‘what kind of ethically challenging experiences have you had in clinical midwifery care practice?’ The semi-structured interviews lasted for an average of 1 h with some individual time variances and were achieved once. The data collection was conducted in the participant’s education institution settings, recorded and transcribed to written text.

The interview guide themes comprised questions regarding the participants’ perceptions of ethical competence in clinical midwifery care and questions such as how students performed their achieved ethical competence in the clinical field of midwifery care practice when the ethically challenging situations occurred? and how education institutions who train midwives should further support midwifery students’ ethical competence?

## The hermeneutical interpretative process

In this study, the hermeneutical interpretation originates in Gadamer’s philosophy, Eriksson’s caring science tradition and the study’s research questions. The semi-structured focus group interviews with midwifery students were a form of narratives that focused on a specific phenomenon: to achieve an understanding of the substance of the midwifery students’ lived experiences of support for ethical competence in clinical midwifery care practice expressed through the participants’ narratives.^33,34^

Gadamer’s^17^ hermeneutical spiral’s circular movement comprises questions and answers and describes the universal whole-part interpretations. Through interpretation or tradition, it changes the understanding and outlines new horizons of understanding about the explored study object. Understanding brings together different and previously separated horizons. The universal parts of the interpretation of this study are transformed into text that moves on the abstract levels of the hermeneutical interpretation process that intertwine. Increasingly, a comprehensive understanding of the meaning of the text could be obtained. Gadamer’s^17^ hermeneutical circle provides the researcher the ability to perform a dialectic movement between understanding and explanation.

The interpretative process and the text from the narratives were approached throughout with openness and respect for the midwifery students and their experienced lifeworld.^17,33^ The texts were re-read several times to delve more deeply into the text. During the hermeneutical interpretative process, the focus was directed towards the following questions: what is the text saying? what is the actual meaning with the text? and what are the implications imparted by the text? The researchers agreed to challenge own prejudices and pre-understandings, and at the same time protected the narrative texts to be manoeuvred from the hermeneutical interpretation process. Through the circular movements between the parts and the whole, substantial phases of expressions and citations of meaningful texts were separated, organised, reorganised and systematised into various hermeneutic thematic themes. These themes describe meaningful narrative texts from the different focus groups. In the last interpretative movement, the text was read once more as a whole, and the new understanding and meaning units were described in relation to the text.^17,33,35^

## Ethical considerations

**Figure 1.**
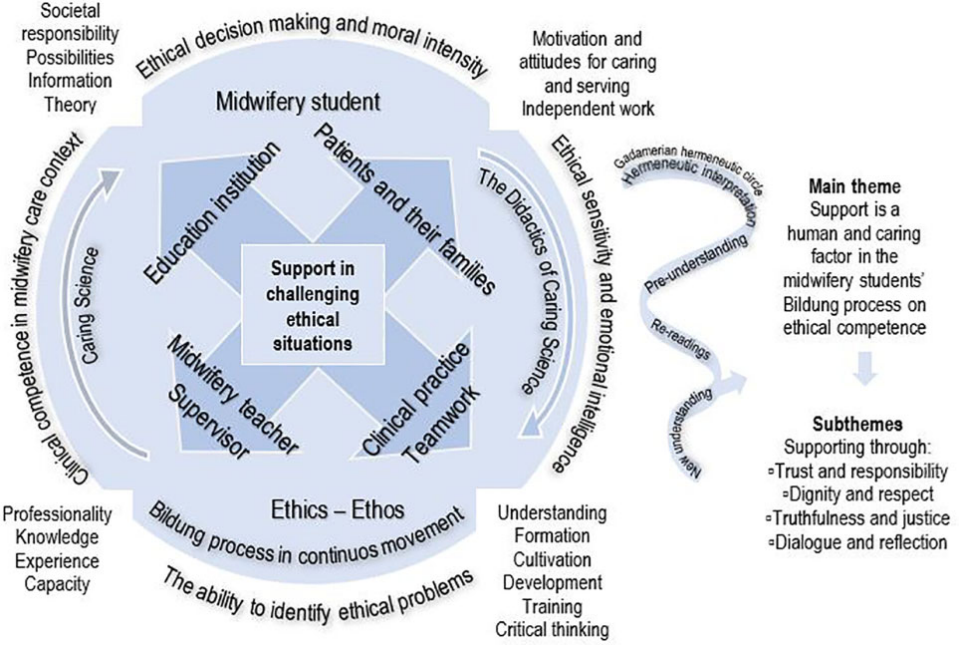
Education institutions’ important mission is to support the midwifery students’ ethical Bildung process on the demands of clinical practice and the becoming working life development, caring science research and the midwifery care cultural points of departue. The Gadamerian hermeneutic circle describes the whole-part interpretations of understanding in the context of midwifery students in the clinical midwifery care context. One main theme and four subthemes were created from hermeneutical interpretations from midwifery students’ narratives.

The study was performed in accordance with accepted research ethical standards, guidelines and codes.^37^ An approval from an ethical board was not necessary. This is in agreement with the Swedish ethical guidelines and laws.^38^ Participants received oral and written information about the study and the possibility to withdraw without justification. They signed an informed consent prior to inclusion. Participation was voluntary and anonymous. Publishing the study results is the last ethical question in all research and linked to legitimacy. This means that the results can benefit individual humans and the society as well as the cause of health and the caring science (Figure 1).^35^

## Results

The results are presented according to the study objective and research questions, narratives from midwifery students and a hermeneutic interpretation of the findings. The results are related to the chosen theoretical perspective and previous research. The use of concepts and descriptions was equal among the participants. One main theme and four subthemes were created from the interpretation. The main theme was as follows: support is a human and caring factor in the midwifery students’ Bildung process on ethical competence. The subthemes were as follows: supporting through trust and responsibility; supporting through dignity and respect; supporting through truthfulness and justice; and supporting through dialogue and reflection.

### Support is a human and caring factor in the midwifery students’ Bildung process on ethical competence

Obtaining ethical competence in the midwifery education requires support that is human and caring for the midwifery student’s Bildung process emerged as a main theme in this study. The subthemes are intertwined to the continuous Bildung movement, support that is caring and ethical competence in the clinical midwifery care context. The participants explained that ethically challenging situations are most often related to the processing and elucidation of sensitive patient information. Important ethical and humanistic principles should therefore be implemented by supervision and supporting from the earliest stages of the studies. Initiating dialogues and in-depth reflection and exploration on ethical questions opens a new kind of understanding of ethically challenging situations aimed at meeting the good and acting in the best interests of the patient and the family.

In the clinical field, I think that the support and supervision should be associated with an ethical approach…that the person who is teaching or supervising is committed to general ethical values…is professional and competent…

It is important that the support comes even before the ethical problem and that we discuss about it also afterwards…

### Supporting through trust and responsibility

Support is perceived among midwifery students as a positive ethical value that is associated with caring and trust in other persons, such as the midwifery teacher or the clinical supervisor. Midwifery students are also jointly responsible to support each other when challenging ethical situations occur. Receiving support in ethically challenging clinical situations opens for hope and encourages to learn and growth. Trust, hope and the evolving professional identity are unified through midwifery studies. However, trust also includes the feeling of vulnerability that is important to protect.

Trust is a kind of basic ethical orientation or attitude towards this work. If you can trust, you can also see the sensitivity in support…It is possible to get support from the supervisor or the teacher or those at the clinic…especially if you take initiative by yourself…Of course, this is also accompanied by the hope to grow into the best possible midwife…

The possibilities of responsibility in the education are connected to trust, encouraging and enabling…It is important that they trust in us and also give tasks that require responsibility…

### Supporting through dignity and respect

The participants reflected to caring science and professional ethics that ethics affirms human dignity. The midwifery profession is related to the respect of humanity with dignity. Respect for every human gender, sexual orientation and gender diversity should always be connected to the midwifery students’ ethos and ethical manners. In clinical midwifery care practice, the students encounter all kind of human beings in different ages or social status, with different religion or origin. It is necessary that the patients and their families are served with respect, equally, professionally and ethically. This educational learning progress is multifaceted and requires time to be applicated. When the midwifery students by themselves receive caring support, supervision and understanding, they become empowered and are gradually able to function more independently. To reach this ideal phase, the various components of support must act both alone and in relation to each other.

All kinds of people come to the clinic…The college prepares us for it in advance with theoretical studies and supervision…The support and supervision we get, is key…Each of us must be flexible and understand the diversity of different people and put aside our own opinions…It is professionalism and own ethical development…If somebody does not understand this it may be difficult to cope with the studies…

### Supporting through truthfulness and justice

Supporting midwifery students includes a societal responsibility from the educational institution, the trainee hospitals and clinics. Reflecting on ethical challenges in group teaching in theory sessions was perceived as a preparatory support for the practical period. Reflecting on ethical challenges should be understood as a developing and constructive interaction dialogue that is inextricably linked to respect of the human beings’ view, integrity and critical thinking. Support should be informative, fair and consistently promote equality.

Pregnancy abruptions can be ethically complicated…At the same time, you think about those who go through infertility treatments…Then there are these sexual offences too…When such patients come to the clinic, it is important to think carefully about what you say or not say, and think about own body language…Patients are sensitive…Fortunately we discussed these situations in advance before the clinical practice period started…

A sense of situation develops along during the clinical training period…and also the sense about different ethical values and what is important in different moments…

### Supporting through dialogue and reflection

The key components of the midwifery students’ ethical growth and development are related to interaction, dialogue and reflection. The ethical growth and development progresses through midwifery education from the elementary to deeper and more complex. It involves openness to criticism, dialogue, reflection and understanding of the need for support. Midwifery students have right to their own world of values, but in the midwifery care context, the personal ethos and professional responsibilities are tied to the basic task of midwifery work, the prescribed and tightly regulated norms, the educational institutions’ curriculum and societal legislations. The structures of interaction, dialogue and reflection determine the behaviour of midwifery students’ in the clinical midwifery care context, whereby behaviour is not viewed critically but by implementing norms. The interaction should retain the sensitivity to perceive the ethical development of the midwifery students as deeper understanding progresses while listening to morality.

…Sometimes it can be difficult to take into account the different mindsets, identities or individual needs of patients from different cultures. These things can cause ethical challenges…that you think differently…Ethics teaching is mainly oriented towards nurse students and is pretty kind of common…

It feels that specific ethics teaching that is supportive for midwifery students’ is remaining somehow…We study different ethical dilemma cases at the college. It is good since they illuminate the whole and they are ethically evolving…We somehow wonder that where have we learned ethics…the feeling is that the college’s taught theoretical ethical base is not yet enough strong…but yes, it is somehow stuck on us along the way…The world of color has changed from black and white during the study time, and the basic values of various things…We understand now differently…

Discussions with the supervisors are necessary…We students talk to each other at the nurse station or lunchtime. Sometimes we join in a conversation with the clinical staff, by listening and trying to learn from their experiences…This happens especially when some ethically difficult things occur at the clinic…

## Discussion

This hermeneutical study has explored, from the perspective of the didactics of caring science and midwifery students, how support for ethical competence and how ethically challenging situations have been experienced and carried out during midwifery education in clinical midwifery care practice. The results may increase kept knowledge and contribute to new understanding of midwifery students’ experiences on support for ethical competence. The chosen methodological approach has described the research procedure by thematising and interpreting the data material. Open-mindedness, carefulness and systematics have been emphasised to ensure the highest possible validity of the study.^17,33^

The didactics of caring science tradition has guided this study’s humanistic thinking through the theoretical perspective. Eriksson^9^ explains that the hermeneutic approach enables, through understanding and interpretation, to make the ethos that relates to ethics, visible. Understanding and supporting midwifery students in challenging clinical situations develops from ethos that is confirmed in words and activities. The integration of theoretical midwifery studies and clinical practice opens for deeper understanding, change and wider professional ethical competence. According to Matilainen and Eriksson,^16^ the pedagogical and caring science didactical thinking that is rooted in the substance of midwifery education enables the students’ learning and formation. Being curious and open for new knowledge and pedagogical activities gives midwifery students meaning to the didactics of caring science. Matilainen and Eriksson^16^ share as well, von Humboldt’s^18^ idea of Bildung^19^ that is connected with the reflective and uniform nature of intelligence.

The ethics of decision-making that is closely related to clinical ethical competence increases with midwifery education and the experience from clinical practice. Ethical challenges involve moral intensity that affects all stages of ethical decision-making.^2,20,21^ The results of this study show that midwifery students evaluate continuously ethical decisions that are related to clinical midwifery care practice as well as the disadvantages and benefits of the consequences of an individual decision. They weigh from different perspectives what is right or wrong, assess the time distribution between the act and the consequences and predict the probability of the ethical challenge materialising. Facing ethical challenges is versatile and understanding is often based on interpretation. This requires the midwifery students’ ethical sensitivity and emotional intelligence.^17,20,39,40^ The high moral intensity of challenging situations facilitates the identification of ethical signals and necessitates ethical reflection.^20,40^

According to the participants, the study shows that midwifery students are not always aware of the aspect of hidden learning and the continuity of formation on professional ethical competence during their education time. In clinical midwifery care practice, understanding is connected to the willingness to learn and experience from seeing and observing when ethically challenging situations face the students.^7,15,24,29^ Achieving dialogue is united to be interested in, and what is being experienced and therefore also supported. The dialogue provides an opportunity to deepen and increase ethical knowledge and understanding through a completing reflection.^23,40^ Theoretical studies provide a basis for thinking and setting values. The benefits can be seen in clinical care practice when the midwifery students encounter emerging ethical challenges. Ethical values that are focused on midwifery students reflect on a moral-level responsibility on the self, self-development and managing the studies.^23,25,26^

Cannaerts et al.^25^ and also Eby et al.^23^ explain that the important intention in midwifery education is to provide a foundation for ethical competence. Teaching and clinical supervision methods for midwifery students that earlier have been found to be effective and emotionally supportive may no longer be sufficient in the future. Continuous development is needed as the operating environments for healthcare organisations and educational institutions change.^22,29,41^ The development of midwifery services generates new kind of challenging ethical situations that are related to ethical decision-making, where the construction of teaching, clinical supervision and collaboration should be designed to be appropriate to fit in the operating environment of the midwifery context. Despite the changes, multifaceted teaching and clinical supervision is about genuine encounter, equal presence and a caring and supporting professional relationship.^22,23,40^ The foundation for support and understanding is related to the midwifery community’s culture in clinical care practice, where the midwifery students receive learning and formation tasks. Prevailing basic and ethical values as well as caring and support become visible through the space of the existing midwifery community culture.^10,16^

The limitations must be taken into consideration when generalising the results of this qualitative study. The importance of a theoretical perspective in the scientific search for knowledge and its implications for the development of the didactics of caring science is emphasised.^33^ The study involved previous studies which had been carried out in international environments, though the participants were selected from Sweden. The representation of the hermeneutical research method and the focus group interviews, and the combination of methods were limited to the scope for interpreting the material. However, the study is saturated and shows how midwifery students are attending to the support in clinical practice in ethically challenging situations.

This study has explored midwifery students’ experiences of support in challenging ethical situations from a traditional pedagogical viewpoint where teachers and supervisors educate and tutor a particular midwifery student. Tendencies that future studies may develop towards high-fidelity ethics simulation exercises and module practice exist. This will transform the midwifery curriculum, the supervision process and clinical midwifery practice.^12,22,29,41^ This study offers midwifery students’ experiences, substance and a picture of support in challenging ethical situations that can be used as guidance for the didactics of caring science in connection with the planning of theoretical ethics courses and curriculum for midwifery students and supervisor-guided clinical supervision. It is understandable that there exists a constant expectation for developing strategies and procedures based on caring science research and interdisciplinary collaboration.

## Conclusion

Midwifery students’ formation towards ethical competence is connected to a caring culture that permeates ethos, caring support and understanding from the educational institution and healthcare organisations, where the students meet the challenging world of clinical midwifery care. Midwifery students experience that support to grow and develop ethical competence generally takes place. The clinical practice period advances midwifery students’ world of values and its reflection as well as working in ethical situations that are challenging. The study opens for the view that the handling of ethical dilemma may remain theoretical if the midwifery students do not gain personal experience for the formation of challenging ethical situations.
